# Endotracheal tube cuff pressure in three hospitals, and the volume required to produce an appropriate cuff pressure

**DOI:** 10.1186/1471-2253-4-8

**Published:** 2004-11-29

**Authors:** Papiya Sengupta, Daniel I Sessler, Paul Maglinger, Spencer Wells, Alicia Vogt, Jaleel Durrani, Anupama Wadhwa

**Affiliations:** 1 Outcomes Research™ Institute, University of Louisville, 501 E. Broadway, Suite 210, Louisville, KY 40202, USA; 2 Department of Anesthesiology and Perioperative Medicine, University of Louisville, 530 S. Jackson St. University Hospital, Louisville, KY 40202, USA; 3 School of Medicine, University of Louisville School of Medicine, Louisville, KY 40292, USA

## Abstract

**Background:**

Cuff pressure in endotracheal (ET) tubes should be in the range of 20–30 cm H_2_O. We tested the hypothesis that the tube cuff is inadequately inflated when manometers are not used.

**Methods:**

With IRB approval, we studied 93 patients under general anesthesia with an ET tube in place in one teaching and two private hospitals. Anesthetists were blinded to study purpose. Cuff pressure in tube sizes 7.0 to 8.5 mm was evaluated 60 min after induction of general anesthesia using a manometer connected to the cuff pilot balloon. Nitrous oxide was disallowed. After deflating the cuff, we reinflated it in 0.5-ml increments until pressure was 20 cmH_2_O.

**Results:**

Neither patient morphometrics, institution, experience of anesthesia provider, nor tube size influenced measured cuff pressure (35.3 ± 21.6 cmH_2_O). Only 27% of pressures were within 20–30 cmH_2_O; 27% exceeded 40 cmH_2_O. Although it varied considerably, the amount of air required to achieve a cuff pressure of 20 cmH_2_O was similar with each tube size.

**Conclusion:**

We recommend that ET cuff pressure be set and monitored with a manometer.

## Background

A critical function of the endotracheal tube cuff is to seal the airway, thus preventing aspiration of pharyngeal contents into the trachea and to ensure that there are no leaks past the cuff during positive pressure ventilation. However, complications have been associated with insufficient cuff inflation. Consequences of micro-aspiration of oropharyngeal secretions include nosocomial pulmonary infections [1]. Conventional high-volume, low-pressure cuffs may not prevent micro-aspiration even at cuff pressures up to 60 cm H_2_O [2], although some studies suggest that only 25 cm H_2_O is sufficient [3]. In contrast, newer ultra-thin cuff membranes made from polyurethane effectively prevent liquid flow around cuffs inflated only to 15 cm H_2_O [2]. In the absence of clear guidelines, many clinicians consider 20 cm H_2_O a reasonable lower limit for cuff pressure in adults.

Catastrophic consequences of endotracheal tube cuff over-inflation such as rupture of the trachea [4-6], tracheo-carotid artery erosion [7], and tracheal innominate artery fistulas are rare now that low-pressure, high-volume cuffs are used routinely. However, post-intubation sore throat is a common side effect of general anesthetic and may partly result from ischemia of the oropharyngeal and tracheal mucosa [8-10], and the most common etiology of non-malignant tracheoesophageal fistula remains cuff-related tracheal injury [11,12]. In addition, acquired laryngeal stenosis may be caused by mechanical abrasion or pressure necrosis of the laryngeal mucosa secondary to high cuff pressure [13,14]. Animal data indicate that a cuff pressure of only 20 cm H_2_O may significantly reduce tracheal blood flow with normal blood pressure and critically reduces it during severe hypotension [15]. Similarly, inflation of endotracheal tube cuffs to 20 cm H_2_O for just four hours produces serious ciliary damage that persists for at least three days [16]. Thus, appropriate inflation of endotracheal tube cuff is obviously important.

Lomholt et al. recommended selecting a cuff pressure of 25 cmH_2_O as a safe minimum cuff pressure to prevent aspiration and leaks past the cuff [17]; Bernhard et al. supported this recommendation [18]. On the other hand, Nordin et al. studied the relationship between cuff pressure and capillary perfusion of the rabbit tracheal mucosa and recommended that cuff pressure be kept below 27 cm H_2_O (20 mmHg) [19]. Seegobin and Hasselt reached similar conclusions in an *in vitro *study and recommended cuff inflation pressure not exceed 30 cm H_2_O [20]. It is thus essential to maintain cuff pressures in the range of 20–30 cm of H_2_O

Adequacy of cuff inflation is conventionally determined by palpation of the external balloon. Previous studies suggest that this approach is unreliable [21,22]. One study, for instance, found that cuff pressure exceeded 40 cm H_2_O in 40-to-90% of tested patients [22]. However, increased awareness of over-inflation risks may have improved recent clinical practice. Our first goal was thus to determine if cuff pressure was within the recommended range of 20–30 cmH_2_O, when inflated using the palpation method. Because cuff inflation practices are likely to differ among clinical environments, we evaluated cuff pressure in three different practice settings: an academic university hospital and two private hospitals. It is also likely that cuff inflation practices differ among providers. We therefore also evaluated cuff pressure during anesthesia provided by certified registered nurse anesthetists (CRNAs), anesthesia residents, and anesthesia faculty.

Cuff pressure can be easily measured with a small aneroid manometer [23], but this device is not widely available in the United States. It would thus be helpful for clinicians to know how much air must be injected into the cuff to produce the minimum adequate pressure. We designed this study to observe the practices of anesthesia providers and then determine the volume of air required to optimize the cuff pressure to 20 cmH_2_O for various sizes of endotracheal tubes. Taking another approach to the same question, we also determined compliance of the cuff-trachea system *in vivo *by plotting measured cuff pressure against cuff volume.

## Methods

With approval of the University of Louisville Human Studies Committee and informed consent, we recruited 93 patients (42 men and 51 women) undergoing elective surgery with general endotracheal anesthesia from three hospitals in Louisville, Kentucky: 41 patients from University Hospital (an academic centre), 32 from Jewish Hospital (a private hospital), and 20 from Norton Hospital (also a private hospital).

Patients with emergency intubations, difficult intubations, or intubation performed by non-anesthesiology staff; pregnant women; patients with higher risk for aspiration (e.g., full stomach, history of reflux, etc.); and patients with known anatomical laryngeo-tracheal abnormalities were excluded from this study. The Human Studies Committee did not require consent from participating anesthesia providers. However, no data were recorded that would link the study results to specific providers.

### Protocol

Independent anesthesia groups at the three participating hospitals provided anesthesia to the participating patients. Because one purpose of our study was to measure pressure in the endotracheal tube cuff during routine practice, anesthesia providers were blinded to the nature of the study. They were only informed about the second purpose of the study: determining the relationship between cuff volume and pressure. Ninety-three patients were randomly assigned to the study. The groups were not equal for the three different types of practitioners; however, determining differences of practice between different anesthesia providers was not the primary purpose of our study.

General anesthesia was induced by intravenous bolus of induction agents, and paralysis was achieved with succinylcholine or a non-depolarizing muscle relaxant. Male patients were intubated with an 8 or 8.5 mm internal diameter endotracheal tube, and female patients were intubated with a 7 or 7.5 mm internal diameter endotracheal tube. This is a standard practice at these hospitals. Patients who were intubated with sizes other than these were excluded from the study. Anesthesia was maintained with a volatile aesthetic in a combination of air and oxygen; nitrous oxide was not used during the study period.

At the University of Louisville Hospital, at least 10 patients were evaluated with each endotracheal tube size (7, 7.5, 8, or 8.5 mm inner diameter [Intermediate Hi-Lo^® ^Tracheal Tube, Mallinckrodt, St. Louis, MO]); at Jewish Hospital, at least 10 patients each were evaluated with size 7, 7.5, and 8 mm Mallinckrodt Intermediate Hi-Lo^® ^Tracheal Tubes; and at Norton Hospital, 10 patients each were evaluated with size 7 and 8-mm Mallinckrodt Intermediate Hi-Lo^® ^Tracheal Tubes. Consecutive available patients were enrolled until we had recruited at least 10 patients for each endotracheal tube size at each participating hospital. All tubes had high-volume, low-pressure cuffs.

### Measurements

We recorded endotracheal tube size and morphometric characteristics including age, sex, height, and weight.

An anesthesia provider inserted the endotracheal tubes, and the intubator or the circulating registered nurse inflated the cuff. This is the routine practice in all three hospitals. Adequacy is generally checked by palpation of the pilot balloon and sometimes readjusted by the intubator by inflating just enough to stop an audible leak. Investigators measured the cuff pressure at 60 minutes after induction of anesthesia using a manometer (VBM, Sulz, Germany) that was connected to the pilot balloon of the endotracheal tube cuff via a three-way stopcock. This type of aneroid manometer is nearly as accurate as a mercury manometer, but easier to use [23]. Pressure was recorded at end-expiration after ensuring that the patient was paralyzed. The cuff pressure was measured once in each patient at 60 minutes after intubation. We did not collect data on the readjustment by the providers after intubation during this hour.

A syringe attached to the third limb of the stopcock was then used to completely deflate the cuff, and the volume of air removed was recorded. The cuff was considered empty when no more air could be removed on aspiration with a syringe. The cuff was then progressively inflated by injecting air in 0.5-ml increments until a cuff pressure of 20 cmH_2_O was achieved. The entire process required about a minute.

### Data analysis

Our primary outcomes were 1) measured endotracheal tube cuff pressures as a function of tube size, provider, and hospital; and 2) the volume of air required to produce a cuff pressure of 20 cmH_2_O as a function of tube size. Outcomes were compared by tube size, provider, and hospital with either an ANOVA (if the values were normally distributed) or the Kruskal-Wallis statistic (if the values were skewed). Compliance of the cuff system was evaluated by linear regression of measured cuff pressure *vs*. measured cuff volume. Data are presented as means (SD) or medians [interquartile ranges] unless otherwise noted; *P *< 0.05 was considered statistically significant.

## Results

Morphometric and demographic characteristics of the patients were similar at each participating hospital (Table 1). Measured cuff pressures averaged 35.3(21.6)cmH_2_O; only 27% of the patients had measured pressures within the recommended range of 20–30 cmH_2_O. Fifty percent of the values exceeded 30 cmH_2_O, and 27% of the measured pressures exceeded 40 cmH_2_O. Thus, 23% of the measured cuff pressures were less than 20 mmHg. Measured cuff volume averaged 4.4 ± 1.8 ml. Neither measured cuff pressure nor measured cuff volume differed among the hospitals (Table 2).

**Table 1:** Demographic and Morphometric Characteristics as a Function of Hospital.

	**University of Louisville Hospital**	**Norton Hospital**	**Jewish Hospital**	***P***
**N**	41	20	32	---
**Tube size (N) 7 / 7.5 / 8 / 8.5**	10 / 11 / 10 / 10	10 / 0 / 10 / 0	10 / 10 / 12 / 0	---
**Age (yr)**	41 (14)	50 (14)	51 (16)	0.006
**Weight (kg)**	88 (27)	100 (40)	78 (17)	0.020
**Height (m)**	1.7 (0.1)	1.7 (0.1)	1.7 (0.1)	0.669

Results presented as number or mean (SD).

**Table 2:** Principal Results as a Function Hospital

	**University of Louisville Hospital**	**Norton Hospital**	**Jewish Hospital**	***P***
**Measured Cuff Pressure (cmH_2_O)**	26 [18, 38]	34.5 [20, 50.5]	33 [22.5, 48.5]	0.469
**Measured Cuff Volume (ml)**	4.0 [3.0, 5.0]	4.3 [3.0, 6.0]	4.5 [3.2, 5.5]	0.646
**Volume Required for 20 cmH_2_O (ml)**	2.7 [2.1, 3.8]	2.5 [2.3, 3.3]	2.9 [2.2, 3.7]	0.792

Results presented as median [interquartile range].

There was no correlation between the measured cuff pressure and the age, sex, height, or weight of the patients. Nor did measured cuff pressure differ as a function of endotracheal tube size. Measured cuff volumes were also similar with each tube size. Interestingly, the amount of air required to achieve a cuff pressure of 20 cmH_2_O was similar with each tube size (Table 3). However, there was considerable variability in the amount of air required.

**Table 3:** Principal Results as a Function of Tube Size.

	**7.0 mm**	**7.5 mm**	**8.0 mm**	**8.5 mm**	***P***
**Patients with measured cuff pressure >30 cmH_2_O (%)**	57	57	47	30	0.444
**Measured Cuff Pressure (mmHg)**	32 [22, 52]	38 [24, 49]	30 [16, 38]	24 [21, 40]	0.467
**Measured Cuff Volume (ml)**	3.9 [3.0, 5.0]	4.5 [2.7, 5.0]	4.6 [3.5, 6.1]	3.8 [3.0, 5.0]	0.616
**Volume Required for 20 cmH_2_O (ml)**	2.6 [2.0, 3.1]	2.5 [1.8, 3.0]	3.0 [2.5. 4.1]	3.3 [2.0, 3.9]	0.143

Results presented as medians [interquartile range] or percent.

CRNAs (n = 72), anesthesia residents (n = 15), and anesthesia faculty (n = 6) performed the intubations. There were no statistically significant differences in measured cuff pressures among these three practitioner groups (*P *= 0.847).

**Figure 1:**
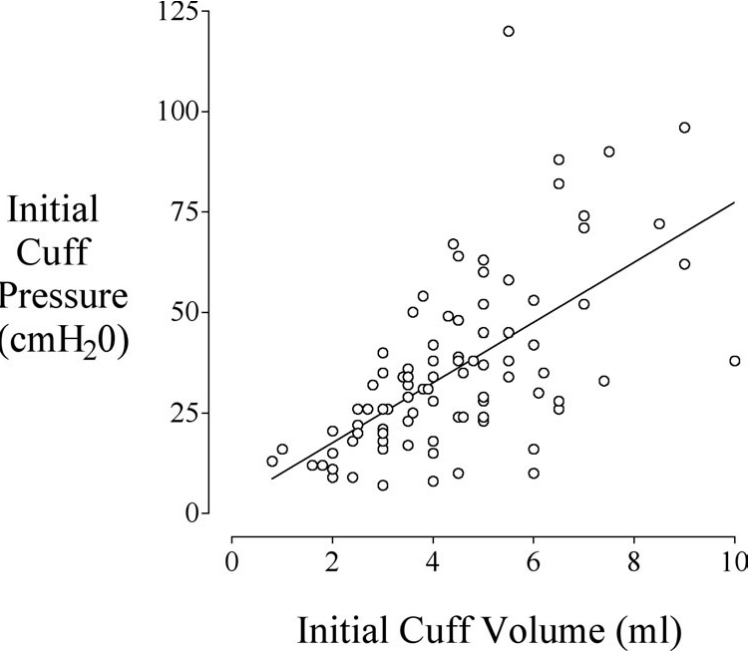
**The relationship between measured cuff pressure and volume of air in the cuff. **There was a linear relationship between measured cuff pressure (cmH_2_O) and volume (ml) of air removed from the cuff: Pressure = 7.5. Volume + 2.7, r^2 ^= 0.39.

The compliance of the tube was determined from the measured cuff pressure (cmH_2_O) and the volume of air (ml) retrieved at complete deflation of the cuff; this showed a linear pressure-volume relationship: Pressure= 7.5. Volume+2.7, r^2 ^= 0.39 (Fig. 1).

## Discussion

Previous studies suggest that the cuff pressure is usually under-estimated by manual palpation. For example, Braz *et al*. [22] observed cuff pressure exceeding 40 cm H_2_O in 91% of PACU patients after anesthesia with nitrous oxide, 55% of ICU patients, and 45% of PACU patients after anesthesia without nitrous oxide. In an experimental study, Fernandez *et al*. [21] observed that when the cuff was inflated randomly to 10, 20, or 30 cmH_2_O, participating physicians and ICU nurses were able to identify the group in 69% of the high-pressure cases, 58% of the normal pressure cases, and 73% of the low pressure cases. Our results are consistent in that measured cuff pressure exceeded 30 cmH_2_O in 50% of patients and were less than 20 cmH_2_O in 23% of patients. Cuff pressures were thus less likely to be within the recommended range (20–30 cmH_2_O) than outside the range. It was nonetheless encouraging that we observed relatively few extremely high values, at least many fewer than reported in previous studies [22]. This result suggests that clinicians are now making reasonable efforts to avoid grossly excessive cuff inflation.

Measured cuff inflation pressures were virtually identical at the three study sites: one academic center and two private hospitals. These data suggest that management of cuff pressure was similar in these two disparate settings. Interestingly, there was also no significant or important difference as a function of provider – measured cuff pressures were virtually identical whether filled by CRNAs, residents, or attending anesthesiologists. Our results thus fail to support the theory that increased training improves cuff management.

We evaluated three different types of anesthesia provider in three different practice settings. Although we were unable to identify any statistically significant or clinically important differences among the sites or providers, our results apply only to the specific sites and providers we evaluated. While it is likely that these results are fairly representative, it is obvious that results would not be identical elsewhere because of regional practice differences.

Fernandez *et al*. [21] found that the volume of air required to inflate the endotracheal tube cuff varies as a function of tube size and type. But interestingly, the volume required to inflate the cuff to a particular pressure was much smaller when the cuff was inflated inside an artificial trachea; furthermore, the difference among tube sizes was minimal under those conditions. We similarly found that the volume of air required to inflate the cuffs to 20 cmH_2_O did not differ significantly as a function of endotracheal tube size. These data suggest that tube size is not an important determinant of appropriate cuff inflation volume. A caveat, though, is that tube sizes were chosen by clinicians in our study and presumably matched patient size; results may well have differed if tube size had been randomly assigned. We intentionally avoided this approach since our purpose was to evaluate cuff pressures and associated volumes in three routine clinical settings.

A limitation of this study is that cuff pressure was evaluated just once 60 minutes after induction of anesthesia. Because nitrous oxide was not used, it is unlikely that the cuff pressures varied much during the first hour of the study cases. We recognize that people other than the anesthesia provider who actually conducted the case often inflated the cuffs. However, a full hour was plenty of time for the provider to have checked and adjusted cuff pressure to a suitable level.

We observed a linear relationship between the measured cuff pressure and the volume of air retrieved from the cuff. The regression equation indicated that injected volumes between 2 and 4 ml usually produce cuff pressures between 20 and 30 cmH_2_O independent of tube size for the same type of tube. However, there was considerable patient-to-patient variability in the required air volume. Measuring actual cuff pressure thus appears preferable to injecting a given volume of air.

## Conclusions

Cuff pressure should be measured with a manometer and, if necessary, corrected.

## Competing interests

The author(s) declare that they have no competing interests.

## Authors' contributions

SP oversaw day-to-day study mechanics, collected data on many of the patients, and wrote an initial draft of manuscript.

DIS contributed to study design, data analysis, and manuscript preparation.

PM, SW, and AV recruited patients and performed many of the measurements.

JD conceived of the study and participated in its design.

AW contributed to protocol development, patient recruitment, and manuscript preparation.

All authors read and approved the final manuscript.

## Pre-publication history

The pre-publication history for this paper can be accessed here:


